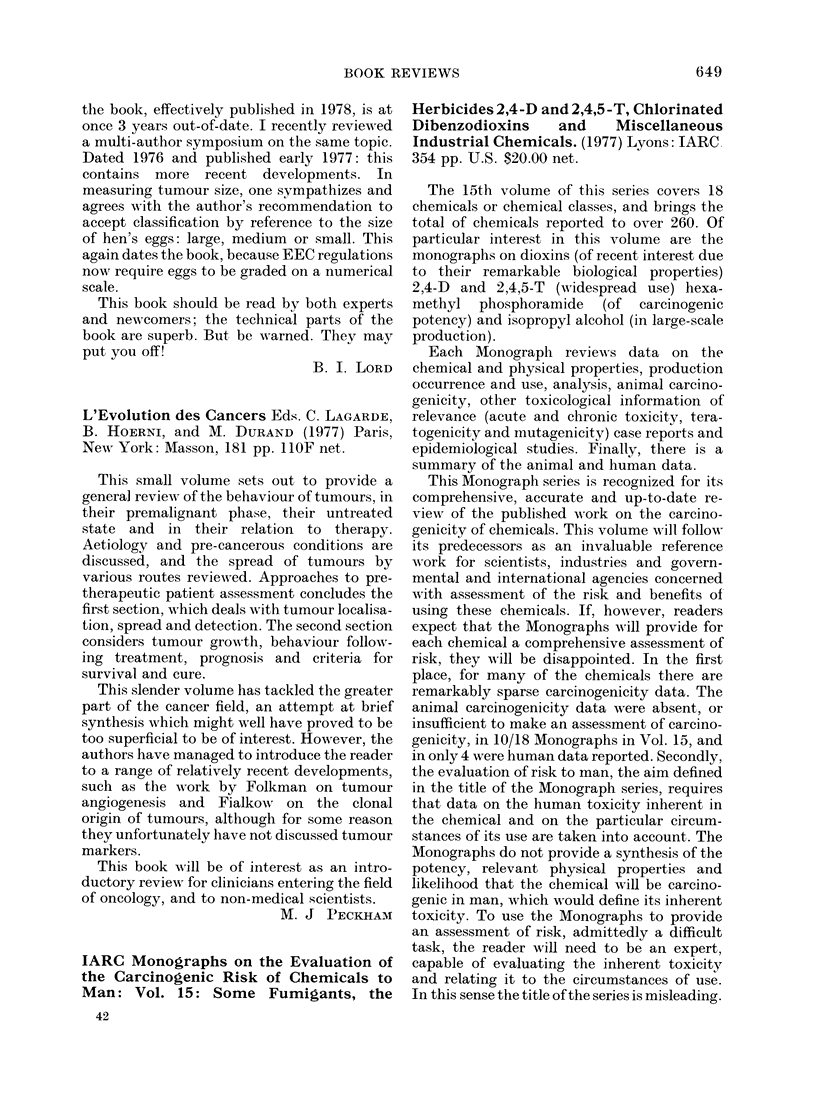# L'Evolution des Cancers

**Published:** 1978-04

**Authors:** M. J Peckham


**L'Evolution des Cancers** Eds. C. LAGARDE, B. HOERNI, and M. DURAND (1977) Paris, New York: Masson, 181 pp. 110F net.

This small volume sets out to provide a general review of the behaviour of tumours, in their premalignant phase, their untreated state and in their relation to therapy. Aetiology and pre-cancerous conditions are discussed, and the spread of tumours by various routes reviewed. Approaches to pre-therapeutic patient assessment concludes the first section, which deals with tumour localisation, spread and detection. The second section considers tumour growth, behaviour following treatment, prognosis and criteria for survival and cure.

This slender volume has tackled the greater part of the cancer field, an attempt at brief synthesis which might well have proved to be too superficial to be of interest. However, the authors have managed to introduce the reader to a range of relatively recent developments, such as the work by Folkman on tumour angiogenesis and Fialkow on the clonal origin of tumours, although for some reason they unfortunately have not discussed tumour markers.

This book will be of interest as an introductory review for clinicians entering the field of oncology, and to non-medical scientists.

M. J PECKHAM